# WDM Network and Multicasting Protocol Strategies

**DOI:** 10.1155/2014/581052

**Published:** 2014-03-12

**Authors:** Pinar Kirci, Abdul Halim Zaim

**Affiliations:** ^1^Computer Engineering Department, Istanbul University, 34010 Istanbul, Turkey; ^2^Computer Engineering Department, Istanbul Commerce University, 34840 Istanbul, Turkey

## Abstract

Optical technology gains extensive attention and ever increasing improvement because of the huge amount of network traffic caused by the growing number of internet users and their rising demands. However, with wavelength division multiplexing (WDM), it is easier to take the advantage of optical networks and optical burst switching (OBS) and to construct WDM networks with low delay rates and better data transparency these technologies are the best choices. Furthermore, multicasting in WDM is an urgent solution for bandwidth-intensive applications. In the paper, a new multicasting protocol with OBS is proposed. The protocol depends on a leaf initiated structure. The network is composed of source, ingress switches, intermediate switches, edge switches, and client nodes. The performance of the protocol is examined with Just Enough Time (JET) and Just In Time (JIT) reservation protocols. Also, the paper involves most of the recent advances about WDM multicasting in optical networks. WDM multicasting in optical networks is given as three common subtitles: Broadcast and-select networks, wavelength-routed networks, and OBS networks. Also, in the paper, multicast routing protocols are briefly summarized and optical burst switched WDM networks are investigated with the proposed multicast schemes.

## 1. Introduction

In our century, internet is an indispensable technology and the main reason of the increasing internet traffic is the ever growing demands of the users. Thus, new technologies are needed for high-bandwidth intensive applications to provide enough bandwidth. WDM optical network is proposed to deal with this problem. Optical network occupies local to wide area, connects millions of users, and offers high data rates and capacities exceeding those of familiar networks. WDM technology well utilizes the high-bandwidth characteristic of the fiber-optic links. In the concept of WDM over fiber-optic link, the laser beams travel over a single fiber where each laser beam travels over a different optical wavelength [1]. Hence, on these days, WDM networks become the most preferred architecture for backbone networks with the many advantages they own. The first one is the ability of building robust multicast trees with the optical layer topology instead of electronic layer. The other one is the ability of light-splitting which is more efficient than copying IP packets. The last one is the bit-rate transparency in optical multicast. Furthermore, multicast services' needed applications are also gaining great concern. Multicasting is a way of sending information from a single or multiple sources to many destinations. Recently, multicasting in optical domain gains much more attention than multicasting in electronic domain because of the light-splitting capability of optical switches and because these optical switches present excellent solutions instead of duplicating data in electronic domain [2–4].

The paper is organised as follows. In Section 2, WDM networks' general structure is presented. Optical WDM networks and main topics about multicasting are briefly summarized in Section 3. In the last section, a new multicasting protocol for OBS is proposed.

## 2. General Properties of WDM Networks

As the bandwidth intensive application usage increases, the bandwidth demand increases too. Therefore, WDM is presented to meet this increasing need for very-high-bandwidth transport networks. With the WDM technology, it is easy to build very large wide-area networks. Thus, internet, video-on-demand, distributed interactive simulation, graphics and visualization, worldwide web browsing, e-commerce, medical image access and distribution, shared whiteboards, teleconferencing, and many more bandwidth intensive applications demand multicast services. On the other hand, these multicast applications belong to the category of multipoint applications.

The first service is* one-to-many* which includes most of the applications such as on-demand video distribution, network news distribution, file distribution, and document distribution. The second service,* many to one,* includes resource discovery, data collection at a central location, auctions, group polling, and accounting. The last service* many to many* applications are multimedia conferencing, distance learning, and distributed simulations. The transmission rates of these applications are on the order of subwavelength rates. This means that some of the applications do not need the whole transmission rates of a lightpath. Therefore, for this kind of applications, traffic grooming is required. In the traffic grooming technique, different traffic streams are switched into higher-speed streams. There are many traffic grooming models in the literature but most of them are designed for unicast traffic. It is obvious that the network design and traffic structures of unicast traffic and a multicast traffic are different from each other. For example, in order to support a multicast traffic, the packets need to be duplicated but in a unicast traffic there is no need for packet duplication. In unicast connections, a path should be routed from source to destination. Similarly, in multicast connections, the optical signal should be routed from the source of a tree and span all destinations. Also, wavelength assignment should be done for these connections. The problem of assigning wavelengths and routes for a group of connections is known as routing and wavelength assignment (RWA) [1, 3, 4]. Multicast routing and wavelength assignment (MC-RWA) problem is studied according to many aspects. Some of these aspects are proposed in [5] with the RWA problem investigation.

The main structure of an optical WDM is composed of many routing nodes that support a definite number of wavelengths. Also, these nodes are connected to each other by point-to-point fiber links. In a WDM network, a connection is named as light path. In general, there is a packet transmission from one source to multiple destinations in a multicast network. For this type of transmissions there should be a connection established from one source node to many destination nodes. 

For an efficient multicasting, it is better for the nodes to have the capability of splitting and/or broadcasting from input to output. In some situations, an incoming channel is needed to be connected to a group of outgoing channels each of which is on a different fiber. There are three multicast models. In* multicast with same wavelength* (MSW) model, the same wavelength is assigned to the source and the whole destinations in the multicast connection; that is, there is no wavelength conversion taking place in the transmission. In the* multicast with same destination wavelength* (MSDW) model, the same wavelength is assigned to the whole output signals but this wavelength may be different from the input wavelength. The last model is* multicast with any wavelength* (MAW). In this model, the source and destinations use different wavelengths [6].

These types of network services require optical cross-connects (OXC) to be able to switch data carrying connections. In general, opaque (optical-electronic-optical) and transparent (all-optical) switching architectures are studied with the fractional capacity sessions and sparse splitting constraints [7].

**Figure 1:**
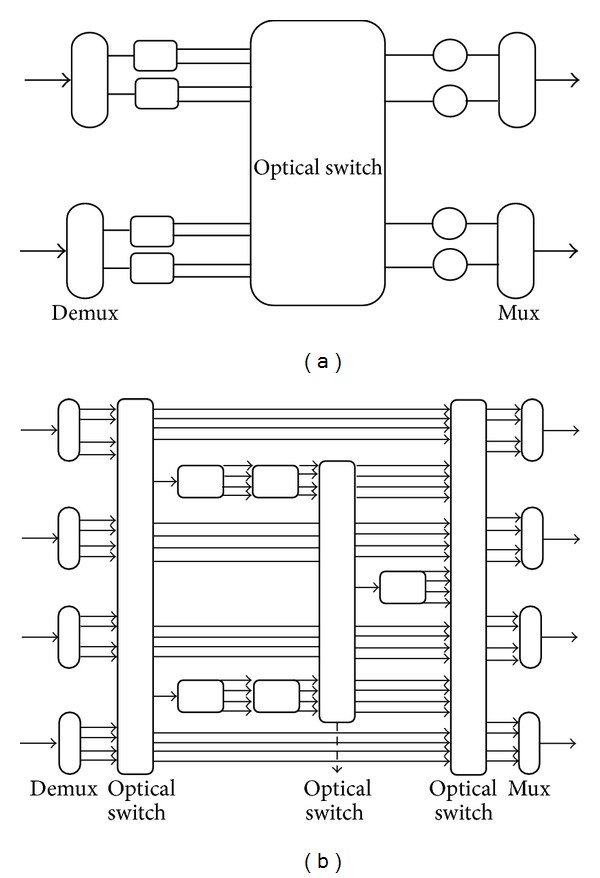
(a) 2 × 2 switch architecture and (b) 4 × 4 switch architecture.

Clearly, optical switches are essential for WDM networks. The switches may have much different architecture and two of them are shown in Figure 1. They have two important facilities; these are routing of optical paths and termination or addition of optical paths. Light-splitting capability is so important for optical switches in bandwidth intensive applications. In all-optical networks, power is such an important measurement. For this reason, in optical multicasting, power splitters need to be used. These devices are distributing the input signal to all outputs so there is no need for buffering in multicast connections [8].

The basic architectures of WDM networks in literature are summarized with three sub titles. The general structure of a broadcast-and-select WDM network consists of *N* nodes, a passive star coupler (PSC), and optical fibers. The nodes and passive star couplers are connected to each other with a pair of optical fibers. Also, the nodes have a number of transmitters and receivers. The transceivers may be fixed to a wavelength or not. If they are not fixed, then they are tunable over a number of wavelengths. In literature, there are four types of node transceivers explained. These are fixed transmitter and fixed receiver (FT-FR), fixed transmitter and tunable receiver (FT-TR), tunable transmitter and fixed receiver (TT-FR), and tunable transmitter and tunable receiver (TT-TR).

Broadcast-and-select in WDM networks can be examined in two categories. These are single hop and multihop. In a single hop network, in order to support a packet transmission, the source node's transmitter and the destination node's receiver should be tuned to the same wavelength. When a source node sends a data, it passes through the PSC, but not an intermediate node, and reaches the destination node. In general, broadcast-and-select networks utilize PSCs. Here, every node is connected to a passive star with two-way fibers. In this system, the data is transmitted from *N* different nodes to passive star via *N* wavelengths. Furthermore, the nodes have only one receiver which is tuned to only one of the *N* channels and also these nodes may listen to only one of the *N* information streams. In other words, a PSC is used for dividing the incoming light from any port equally to all other ports. Hence, the arriving packet is broadcasted to all of the other ports by a PSC. When a node receives a packet, it checks if this packet belongs to itself or not. Thus, single hop WDM network is a kind of shared-channel network. Here, the system utilizes wavelength-agile transmitters or receivers, which can tune quickly to different wavelengths but, as mentioned before, this function costs higher than multihop communication. Compared with the multihop networks, single hop networks need expensive wavelength tunable transceivers. The observations imply that these kinds of networks may have contentions during the packet transmissions. Collision is a type of contention, it occurs when two or more transmitters try to transmit to the same destination node with tunable receivers over different channels simultaneously. To avoid this type of contentions, the most important fact is the coordination of transmissions which is adjusted by a media access protocol or a multicast scheduling algorithm (MSA).

There are three types of well-known multicast scheduling algorithms. The first one is reservation-based MSA. Here, a shared control channel is utilized for sending nodes' transmission requests. The arriving packets are placed in an arrival queue and a control packet is sent over a control channel to all of the nodes. The control channel has a slotted structure and the access of the nodes are arranged by round-robin time division multiple access (TDMA). In fact, the control channel is divided into control frames and each of these frames is also subdivided into *N* control slots. If node *i* wants to transmit its control packet, it can only transmit the packet in the *i*th slot of each control frame. When the transmission issue is over, then the data packet is sent to the nodes' waiting queue, till it is transmitted to all of its own destination nodes. By the way, the control packet will arrive to the nodes in the network after a propagation delay. These nodes will run the same MSA for reserving data channels and time slots and scheduling the transmission of the corresponding packet. Then each node updates its record after the reservation and gets ready for a new scheduling. The second one is random-access-based MSA; Here in order to support the system, a centralized scheduler is used, and the nodes send their requests to the control channel with an unslotted random access protocol but the central scheduling works with a slotted manner. The transmission requests arrive to the centralized scheduler via a control wavelength. After receiving the requests, the centralized scheduler runs the MSA and informs the nodes via a separated control wavelength. In this system, there is a request query for each node which is checked consistently and scheduled to the best slot. This type of MSA is presented to reduce the complexity of reservation-based MSA because the nodes try to access the data channels in a random manner. The third one is preallocation-based MSA which has a static structure. In this system, the transmissions are arranged by predetermined schedules, such as, for each slot, the active transmitters, the corresponding wavelengths, the active receivers are predetermined. Thus, the overhead of the control messages can be decreased.

Besides, multihop WDM network works with cheaper fixed-wavelength transceivers. In other words, it functions with fixed-tuned transmitters and receivers. Furthermore, the network owns a fixed logical topology because of the fixed connectivity of nodes. The main reason of this fact is the FT-FR node structure of the network. The multihop networks' logical topology may be irregular or regular. The irregular topology design has a high routing complexity. Consequently, it may not have gained too much attention. But many of the regular topologies are well studied such as shuffle-net, Manhattan Street (MSN), and Hypercube. Furthermore, the main problem of MC-RWA is finding a multicast routing tree and then allocating necessary wavelengths on every link of the tree. To deal with this problem, it is examined as two subtitles: routing and wavelength assignment. In other words, when a multicast request is received, the first thing is finding a tree-structured route which is rooted at the source and spans all of the destinations. To find such a multicast tree, there are many schemes presented in the literature but the simplest way is utilizing the IP multicast routing protocols at the IP layer. After constructing a tree, the next step is allocating available wavelengths for each link of the tree. Routing and wavelength assignment problems are the main problems of wavelength-routed networks. In the wavelength-routed system, the access nodes have transmitters and receivers. In addition to this, the incoming light is routed to the intended outgoing link by the wavelength routing switches. These switches should be multicast capable of providing multicasting in wavelength-routed networks. In other words, these switches should be able to replicate and forward each incoming optical signal to all outgoing links all optically. In order to support multicasting, it is important for a wavelength-routed switch to have a light-splitting capability. In general, this is achieved by an optical splitter. If a wavelength-routed switch does not have a light-splitting capability, then they are called sparse splitting capable switches. Multicast routing and wavelength assignment (MC-RWA) are basically divided into two categories:* single multicast request* case and* multiple multicast request case*. The first case is also categorized as* multicast tree optimization* and* light-forest construction in the case of sparse splitting*. In fact, the main aim of* single multicast request case* is to find routes from source to all other destinations. In* multicast tree optimization*, all of the nodes in the network are multicast capable; so there are many multicast trees to choose from. In this situation, the best one is chosen but, in* light-forest construction in the case of sparse splitting*, the network has sparse splitting nodes so alternative routes must be found to support multicasting. In order to support MC-RWA, new algorithms are presented for static and dynamic structures. In static structure, the multicast trees are predetermined and cannot be changed till the end of the wavelength assignment, in other words a multicast group's membership does not change during its life time. Also, in the dynamic structure, new multicast trees can be constructed for unserved groups; this means that the nodes may join/leave whenever they want.* Multiple multicast request case* structure is composed of dynamic traffic and static traffic. In dynamic traffic MC-RWA, many multicast requests arrive and leave nodes dynamically. For each arriving request, a route and wavelength should be allocated to decrease the blocking rates. In static traffic MC-RWA, multiple multicast requests arrive statistically and they may be supported simultaneously at a meantime or a long term multicast traffic matrix [7–14].

IP multicast routing protocols are examined in two subtitles which are dense/sparse mode and source-based/core-based modes. The first one depends on the expected distribution of multicast group members throughout the network. The second one depends on the construction method of multicast tree and the root of the multicast tree constructed. The Distance Vector Multicast Routing (DVMRP), the Protocol-Independent Multicasting Dense Mode (PIM-DM), and Multicast Extensions to OSPF (MOSPF) multicast routing protocols are dense mode and also use source-based tree but the well-known multicast routing protocols are the Protocol-Independent Multicasting Sparse Mode (PIM-SM) and the core-based tree (CBT) which are both sparse mode and use shared trees rooted at a core router or rendezvous point (RV) which is a meeting point between sources and destinations of a definite group. Furthermore, PIM-SM may also use source-based tree. DVMRP is the first and most famous routing protocol which is used to support IP multicast on MBone. DVMRP and PIM-DM protocols benefit from a flood and prune algorithm, to form a shorter path tree between the sources and receivers and to discover the exact positions of the receivers.

In DVMRP, a neighbour probe message is sent to the whole network on definite time intervals. The messages include neighbour routers' list that sent their own neighbour probe messages before. So the routers discover their neighbours in the network. DVMRP utilizes a reverse shortest path tree and owns its own integrated unicast counting protocol. Also, tunneling is used to span the nodes which are not multicast capable. In other words, packets are broadcasted to the destinations by Reverse Path Forwarding (RPF). If there is not a downstream member then branches are pruned. Also, if new members on pruned branches want to join the multicast group, then they should send explicit GRAFT messages upstream.

In MOSPF, routers change link state information with each other so they gain the exact and last information about actual state of the network. Hence, periodically multicast group membership is flooded to the routers. As soon as a packet arrives at a router, a shortest path tree is computed and the results are stored for the forwarding of next multicast traffic. MOSPF protocol forwarding model and routing mechanisms are summarized in [15, 16].

The next routing protocol is Protocol-Independent Multicasting (PIM). PIM defines the networks as PIM domains and non-PIM enabled domains. Inside the PIM domains, the routers utilize PIM for multicasting and there are bootstrap routers that distribute the information about rendezvous points. Moreover, on the boundaries of these PIM domains the multicast boundary routers are placed to provide the interaction with the non-PIM enabled domains. PIM includes two different protocols; these are PIM-sparse and PIM-dense.

In PIM-sparse mode, the places of the members are supposed to be a long way off each other and the convenient bandwidth tends to be small. Also, the convenient members should belong to a subnetwork. This routing protocol includes tree structure with rendezvous points. Therefore, it is more suitable for groups which are geographically distributed in the network. Here, to join a group for group membership, explicit join operations are needed. By the way, the data is transmitted to only rendezvous point not to the entire network. PIM-sparse mode structure is suitable for construction of multicast trees with rendezvous points. Rendezvous points are utilized for group awareness and explicit join functions. Additionally, the concept of PIM-dense mode is slightly different from PIM-sparse mode. First of all, the members in the PIM-dense mode are closer to each other and the convenient members might be in every subnetwork. Also, the density of group members is high. Furthermore, it has minimal complexity which is a great advantage for a protocol. According to the PIM-dense mode, the group members of all subnetworks want to receive the data at startup. Thus, flooding and pruning are used. Immediate integrations of new members to the multicast tree are performed by sending graft data units. There are specific differences between PIM-sparse and PIM-dense modes. In PIM-dense mode, periodically transmitted join data units and rendezvous points are not used. Also, PIM-dense mode is preferred to be used in domains but PIM-sparse mode is better for larger networks.

The core-based tree (CBT) includes shared bidirectional multicast trees with rendezvous points. When these trees are constructed, they include current group membership. The concept of CBT structure is to reduce the status information amount and the overhead of control data units. To deal with the status information amount, shared multicast trees are used [17, 18]. Here, the decision about the location of the rendezvous point in the network is important. In literature, many methods are presented for deciding the optimum location. The method in [19] depends on distance vector information to obtain the optimum RP location with the lowest total cost.

## 3. Optical WDM Networks

The main function of WDM structure may be explained as follows. It offers the needed capacity and the connections of the optical devices which are important for performing the transmission in the optical level. At the beginning, WDM technology is presented for increasing the capacity of point-to-point fiber links in opaque networks. For multiple accesses, before the WDM technology, time division multiplexing (TDM) and some hybrid architectures [20] were used. But the great increase about the employment of OXCs in networks inspired the transparent optical network designs. In transparent optical networks, there is no need for signal regeneration or OEO conversions. The main role of an OXC in an optical network is to switch an input fiber's arriving signal to the same wavelength on an output fiber. In literature, the IP traffic in optical WDM networks is transmitted by three main switching technologies. These are optical circuit switching (OCS), optical packet switching (OPS), and optical burst switching (OBS).

In OCS, before starting a transmission, a station-to-station lightpath establishment should be arranged as in telephony technology. After the establishment, the data is transmitted over the lightpath and then the connection is closed so the OCS structure may be defined as three phases: establishment, data transfer, and termination. There is no data loss in OCS but if the traffic load increases, then the packets should wait at the incoming or outgoing queues. OCS switching is also used in high performance computing systems to perform cheaper and power-efficient interconnections but, in OCS, reserving a channel for a connection causes great inefficiency [21–23].

OPS is proposed as a new option for wavelength routing. An OPS node includes an optical switch fabric. The switch fabric is used for the reconfiguration according to the packet headers. The packet headers are either transmitted in-band with the packet or out of band on a separate control channel and processed electronically. It is obvious that processing a header and reconfiguration of a switch takes some time; by the way, the packet needs to be delayed by optical delay lines. Thus, fast switching times are important for optical packet switching. Most of the studies about OPS are about throughput improvement. A study about the throughput analysis with big optical packet switches is presented in [24]. Most of the studies in literature are based on flow control algorithms or the packet size and window size explorations for the improvement of the network throughput. The most important concepts in OPS are routing, traffic control, and error control. In OPS, the packets should be routed from one node to the other one because the source and the destination are not connected directly; also for gaining efficient network performance, the whole network traffic is regulated. Besides, in OPS, the error control is an important feature for preventing the packet loss at the stations.

**Figure 2:**
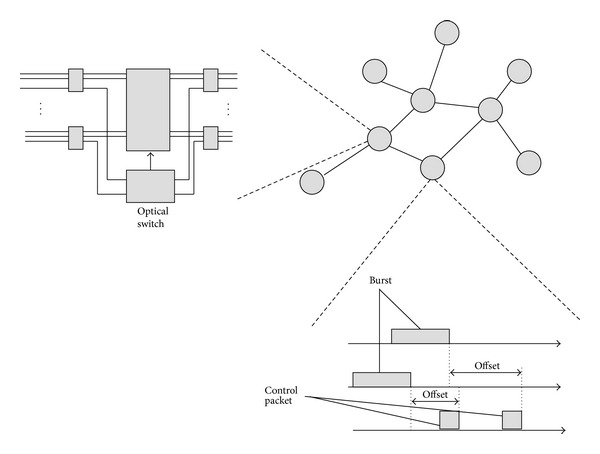
An optical burst switched network.

OBS includes some of the main characteristics of wavelength routing and OPS. In both wavelength routing and OBS, the data is not processed or buffered at the intermediate nodes. Also, in both of the OPS and OBS, the data and the header are processed separately. In OBS, the burst is composed of two parts which are control data and user data. The information carried by the control data are routing information, address information, wavelength channel information and the offset time information. An OBS network is composed of edge routers, core routers, and fiber links. The nodes are connected to each other by fiber links. Moreover, by the help of the WDM technology, these fiber links carry multiple wavelength channels. In ingress edge nodes, first of all, the arriving IP packets are assembled into a burst (called burstification) according to the destination multicast group address and QoS requirements and then scheduled for transmission on outgoing wavelengths. In the core nodes, the bursts are switched from input ports to output ports according to the information in the headers. Furthermore, these core nodes handle burst contentions [25–27]. In an OBS network, multiple IP networks construct bursts and these bursts are switched all-optically. To configure the switches along the bursts' route, a control packet is sent ahead of the burst as presented in Figure 2. During a definite offset time, the control packet is processed. According to the information in the control packet, the switches of intermediate nodes are configured for the coming bursts. In addition to this, the assembled bursts are transmitted over OBS core routers all-optically. First, the bursts arrive at the egress edge node; then the egress edge node disassembles the bursts into packets and then forwards them to the destinations [28–35].

## 4. The Proposed Structure for Optical Burst Switched WDM Networks

In the paper, an optical WDM network is constituted. The network model is composed of multicast capable source, ingress switch, intermediate switches, edge switches, and clients. The nodes of the network are connected to each other by fiber links that carry different numbers of wavelengths.

**Figure 3:**
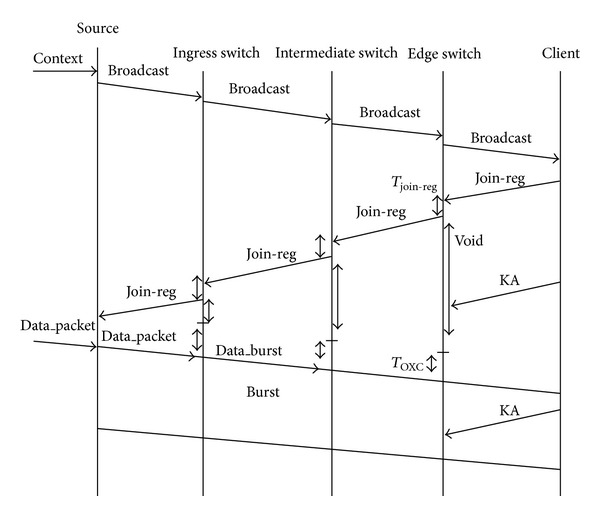
The message flows in the protocol.

The beginning of a session depends on the determined video programs. If a source has a video program to send, it will broadcast the video context to the clients. If the clients want to view the program, they will request the program with sending a join-request message. Consequently, the source multicasts the video bursts to the considered clients. The optimum paths between clients and source are calculated by intermediate switches. Also, the join-request traffic monitoring and controlling is managed by intermediate switches. The request-refused message is sent by the intermediate switch to the related edge switch after receiving a join-request message from the edge switch, to inform the edge switch that it will not be convenient for the video transmission. After receiving the request-refused message, edge switch sends a rejoin-request message to another close intermediate switch for the same video transmission. Also, keepalive messages are sent by clients during the long data transmission periods to inform that the multicasting session connection is still continuing.  Figure 3 demonstrates these basic message flows of the protocol. On the proposed NSFNET topology, all of the switches assumed to have full-splitting capability.

**Figure 4:**
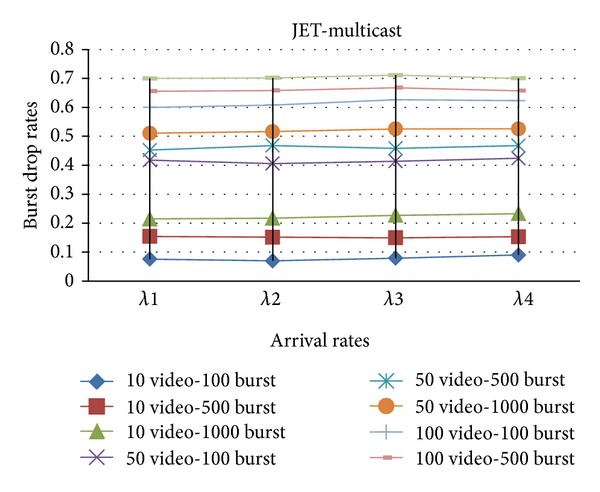
The effect of *λ* values on the burst drop rates with JET.

**Figure 5:**
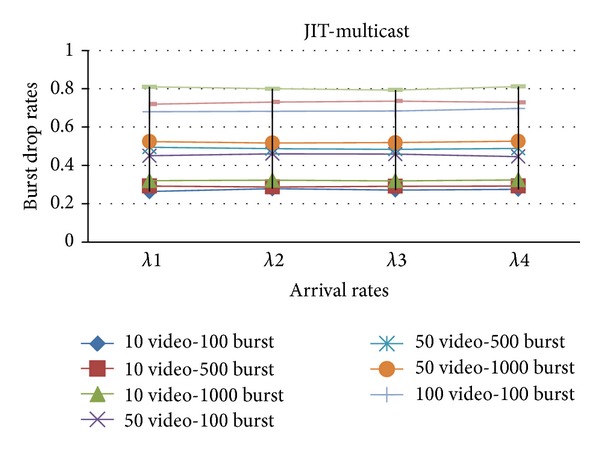
The effect of *λ* values on the burst drop rates with JIT.

In Figures 4 and 5, the simulation is constructed over a network that transmits both multicast and unicast traffic with (Just Enough Time) JET and (Just In Time) JIT reservation protocols on Poisson arrival rates.

**Figure 6:**
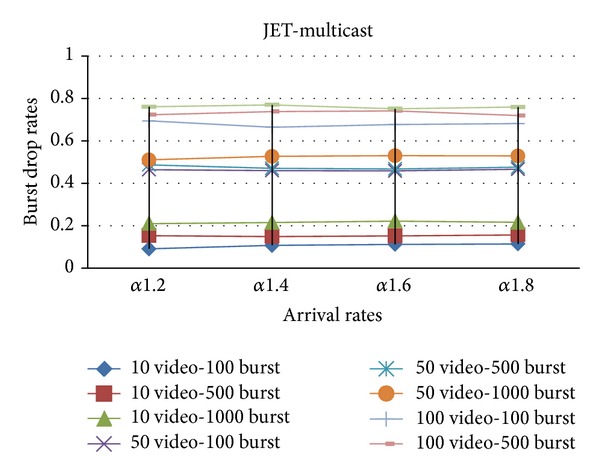
The effect of *α* values on the burst drop rates with JET.

**Figure 7:**
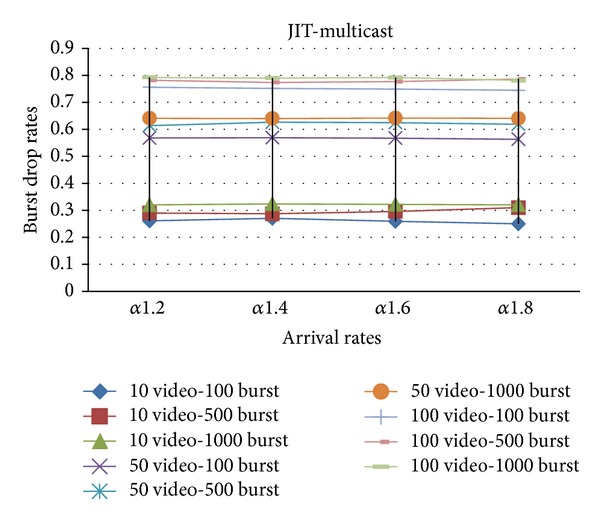
The effect of *α* values on the burst drop rates with JIT.

In Figures 6 and 7, both multicast and unicast traffics are sent over the network with (Just Enough Time) JET and (Just In Time) JIT reservation protocols on self-similar arrival rates.

According to the simulation results, the proposed protocol structure with JET reservation protocol produces less burst drop rates and better performance. By the way, the effect of different arrival rates is also examined in the simulations.

## 5. Conclusion 

Today, the network technologies are in a revolution period. The development of these technologies shapes according to the users' demands and QoS parameters. This paper includes the main and mostly studied topics about WDM networks in literature. In this paper, multicast communications are investigated and core topics are summarized under subtitles. General structure of WDM multicasting is presented with the basic properties of optical switches at the beginning. Broadcast-and-select and wavelength-routed WDM networks are also examined. By the way, IP multicast routing protocols are presented because of their convenient and suitable structures for WDM multicasting. In the following, OBS WDM network structure is given with the other related switching technologies such as OCS and OPS. As we mentioned before, fast optical switches are needed in OPS but, in OBS, the data is transmitted in large bursts; so there is no need for fast optical switches. Also, during the burstification process an OBS core router's switching granularity will be coarser than OPS. Thus, the control and processing overhead decrease. Similarly, OBS provides coarse switching granularity and statistical multiplexing. In other words, OBS combines the advantages of OCS and OPS. As we mentioned before, WDM is a developing technology. Hence, it includes many open research problems. In this paper, we present a general description of WDM networks and multicasting, because we work on a new multicasting protocol on WDM OBS networks and analyze the new structure with different traffic scenarios. Here, a new protocol structure is proposed over OBS network together with performing multicast and considering QoS for real time applications and the structure is supported by simulations. A new multicasting protocol structure is introduced for OBS networks together with the state diagrams and state transmissions of the protocol from source to clients. Over the basic topology, a switch is designed as the traffic source and the clients are the traffic receivers. The burst drop rates are investigated according to their arrivals to the network. Our protocol performance is examined on JET and JIT with multicasting. Our performance results show that the presented protocol structure with JET provides better drop rates than JIT.
